# Simple Biophysical Model Predicts Faster Accumulation of Hybrid Incompatibilities in Small Populations Under Stabilizing Selection

**DOI:** 10.1534/genetics.115.181685

**Published:** 2015-10-03

**Authors:** Bhavin S. Khatri, Richard A. Goldstein

**Affiliations:** * The Francis Crick Institute, Mill Hill Laboratory, London, NW7 1AA, United Kingdom; † Division of Infection and Immunity, University College London, London, WC1E 6BT, United Kingdom

**Keywords:** speciation, Dobzhansky–Muller incompatibilities, sequence entropy, population size, coevolution, genotype–phenotype map

## Abstract

Speciation is fundamental to the process of generating the huge diversity of life on Earth. However, we are yet to have a clear understanding of its molecular-genetic basis. Here, we examine a computational model of reproductive isolation that explicitly incorporates a map from genotype to phenotype based on the biophysics of protein–DNA binding. In particular, we model the binding of a protein transcription factor to a DNA binding site and how their independent coevolution, in a stabilizing fitness landscape, of two allopatric lineages leads to incompatibilities. Complementing our previous coarse-grained theoretical results, our simulations give a new prediction for the monomorphic regime of evolution that smaller populations should develop incompatibilities more quickly. This arises as (1) smaller populations have a greater initial drift load, as there are more sequences that bind poorly than well, so fewer substitutions are needed to reach incompatible regions of phenotype space, and (2) slower divergence when the population size is larger than the inverse of discrete differences in fitness. Further, we find longer sequences develop incompatibilities more quickly at small population sizes, but more slowly at large population sizes. The biophysical model thus represents a robust mechanism of rapid reproductive isolation for small populations and large sequences that does not require peak shifts or positive selection. Finally, we show that the growth of DMIs with time is quadratic for small populations, agreeing with Orr’s model, but nonpower law for large populations, with a form consistent with our previous theoretical results.

SPECIATION is of great importance in generating the observed diversity of life, yet it is still poorly understood, especially at the genetic level. Two populations are said to have speciated when they have developed *reproductive isolation* (RI), that is, when they can no longer interbreed. A standard model of how *postzygotic* reproductive isolation arises is due to Dobzhansky, Muller, and Bateson (Bateson 1909; Dobzhansky 1936; Muller 1942), where so-called Dobzhansky–Muller incompatibilities (DMIs) arise due to epistatic interactions; for example, two geographically isolated lineages evolving allopatrically from a common ancestor *ab* can fix the allelic combinations *aB* and *Ab*, respectively, yet the hybrid genotype AB can be inviable due to the epistatic interactions between these two loci. In polygenic systems, where many loci code for an additive quantitative trait, a similar hybrid incompatibility arises; quadratic, or any nonlinear, selection induces epistasis such that divergent populations, under the action of drift, maintain different underlying allelic combinations at the many loci (Wright 1935a,b) for the same optimal trait value, which when combined in hybrids can lead to incompatibilities (Barton 1989). Although there are many examples of genes directly involved in reproductive isolation (Wu and Ting 2004), we still lack a theoretical understanding of the functional relationship between genes and their role in the development of hybrid incompatibilities and speciation dynamics. In this article, we examine an important example of such a functional relationship, the genotype–phenotype map of transcription factor–DNA binding. Using a simple biophysical model of transcription factor–DNA binding we analyze how incompatibilities can arise between allopatric lineages.

Despite many studies of the evolution of RI, very little attention has been paid to the role of population size; however, there is indirect and direct evidence that smaller populations develop incompatibilities more quickly. The observation of the large diversity of species on small young islands, such as Hawaii (Mayr 1970), or on the island of Cuba (Glor *et al.* 2004) and in the East African Great Lakes (Owen *et al.* 1990; Santos and Salzburger 2012), where in the latter two cases each one has been subject to historically fluctuating water levels and thus opportunities for allopatric speciation, suggests that smaller populations speciate more quickly. This is in contrast to lower levels of reproductive isolation observed in marine species with large ranges and population sizes, for example, the relatively small fraction of Pacific–Caribbean species pairs separated by the Isthmus of Panama a few million years ago compared to those that are not reproductively isolated (Mayr 1954, 1970; Rubinoff and Rubinoff 1971). There is also evidence that reproductive isolation arises more slowly in birds compared to mammals (Fitzpatrick 2004). Strikingly, even after ∼55 MY divergence (Cooper and Penny 1997), domestic chickens (*Gallus gallus*) can still hybridize with helmeted guineafowl (*Numida meleagris*), where estimates of the effective population size of domestic chickens range from $N_{e}\approx 10^{5}$ to $10^{6}$ (Sawai *et al.* 2010), whereas in contrast, cichlids develop reproductive isolation as quickly as 1−10MY after divergence (Stelkens *et al.* 2010) and have relatively small population sizes [100−10,000 (Oppen *et al.* 1997; Fiumera *et al.* 2000)]. This population size trend is further supported by net rates of diversification (Coyne and Orr 2004) inferred from phylogenetic trees (Barraclough and Nee 2001; Nee 2001). On the other hand, there are examples that buck this trend, such as *Drosophila*, which shows rapid speciation, for example, in adaptive radiations in Hawaii at large population size (Ayala *et al.* 1996).

Where does current theory stand in light of these observations? There are a number of theoretical models of allopatric speciation based on the Dobzhansky–Muller mechanism, which consider independent lineages evolving neutrally or under varying selection pressures on each lineage (Nei *et al.* 1983; Orr 1995; Orr and Orr 1996; Orr and Turelli 2001; Gavrilets 1999, 2003, 2004). Models that involve positive selection driving divergence are unlikely to be able to explain this dependence on population size, since larger populations respond more quickly to a given selection pressure (Gavrilets 2003). This leaves models of speciation where populations diverge neutrally or under stabilizing selection pressure; the models of Nei *et al.* (1983) and Gavrilets (1999) tackle precisely this question in the strong mutation regime ($n\mu_{0}N\geq 1,$ where *n* is the number of nucleotides or base pairs for the loci of interest, $\mu_{0}$ the base-pair mutation rate, and *N* the population size) where the population is highly polymorphic. They find slower divergence in larger populations due to the lower reproductive success of members of the population who have diverged farther from the fitness maximum, resulting in a slower speciation rate. However, in neither of these models is there a dependence on population size in the weak mutation, nearly monomorphic regime, where $n\mu_{0}N\ll 1.$ Models of hybrid incompatibility that rely on fitness epistasis on quantitative traits (Wright 1935a,b) also predict that smaller populations should develop reproductive isolation more quickly, as drift helps populations shift between stable equilibria more rapidly (Barton 1989); but again by the polygenic nature of the population described in the model, we expect such a system to have evolutionary dynamics in the strong mutation regime.

A model that could give rise to more rapid RI for small populations is based on founder events or peak shifts, where small founder populations split and become isolated (Lande 1979, 1985; Barton and Charlesworth 1984; Barton and Rouhani 1987); the strength of drift is larger in small populations, allowing them to more easily pass through fitness valleys. A major problem with such models is that for isolation to occur on reasonable timescales the product of the fitness barrier and population size needs to be sufficiently small. However, this condition also means that gene flow is relatively unimpeded between peaks (Coyne and Orr 2004), destroying the reproductive isolation the model seeks to establish. Finally, the work of Orr and co-workers provided a framework to understand how incompatibilities might arise in allopatry through sequentially fixing mutations in the weak mutation regime ($n\mu_{0}N\ll 1$) (Orr 1995; Orr and Turelli 2001); they showed that the number of potential or untested incompatibilities “snowballs” like $∼\;K^{2}$ for interactions between pairs of loci, where *K* is the number of substitutions separating the two lineages. However, the starting point of this model is the assumption of neutral, population size independent, divergence between lineages with a fixed probability that each untested combination is incompatible and so cannot address the question of the population size dependence.

A common theme of the above theories is that they are phenomenological with respect to the underlying genetic basis of incompatibilities. Johnson and Porter (2000, 2007) examined the evolution of decreased hybrid fitness for simple models of gene regulation, under positive and stabilizing selection, in the clonal interference regime ($n\mu_{0}N∼1$), but did not investigate the dependence on population size. More recently, they extended their work with sequence-based models of transcription factor (TF) binding similar to the model described here (Tulchinsky *et al.* 2014b), showing decreased hybrid fitness with decreasing population size; however, these results are again in the regime where the effect of mutations is not weak ($n\mu_{0}N∼1$) and the dynamics of the growth of DMIs were not investigated in detail.

In summary, although the models of Gavrilets, Nei ,and Barton each predict a decreasing rate of developing RI with increasing population size when $n\mu_{0}N\geq 1,$ these models predict no dependence on population size, or are not applicable, in the weak mutation, nearly monomorphic regime where $n\mu_{0}N\ll 1.$ This is despite genetic studies that have shown that traits involved in species differences range from monogenic to mildly polygenic (Orr 2001). However, more recently, a theoretical framework was developed by the authors of this article for phenotypic evolution in the monomorphic regime ($n\mu_{0}N\ll 1$) that accounts for a general mapping between genotype and phenotype (Khatri and Goldstein 2015); when applied to a toy model of transcription factor–DNA binding, it suggested that more rapid RI might arise for smaller populations, due to their having a larger drift load. In this work, we explore simulations of a more realistic sequence-based model of transcription factor–DNA binding, which overcomes limitations of the theoretical model.

Although any pair of interacting genes can result in a DMI, the interaction of genes that control expression has been shown to be a major factor driving differences between species (King and Wilson 1975; Wray 2007; Wittkopp *et al.* 2008; Wolf *et al.* 2010), suggesting a major role in speciation. In particular, compensatory changes at both cis and trans locations have been shown to be responsible for the misexpression of many genes in hybrids between *Drosophila melanogaster* and *D. simulans* (Landry *et al.* 2005), while there is more direct evidence in *Drosophila* of evolution of genes related to transcription factors driving speciation (Ting *et al.* 1998; Brideau *et al.* 2006). With the increasing use of genome-level studies (Seehausen *et al.* 2014) to characterize speciation, there is a need for theory and modeling to bridge the gap between sequence-level changes at coevolving loci and phenotypic determinants of incompatibilities; the binding of transcription factors to DNA to control gene expression is arguably one of the most important coevolving systems for organisms and so makes an ideal case study to examine the consequences to speciation of a simple biophysical model and a mechanistic insight on the way DMIs develop.

In this article, we examine how incompatibilities arise in allopatry for an abstract, yet biophysically motivated model of binding between two macromolecules, a protein TF binding to a specific DNA or TF binding site (TFBS). Our model is based on the “two-state” approximation (Von Hippel and Berg 1986; Gerland *et al.* 2002), which assumes the binding affinity is a sum of contributions of opposing amino acid nucleotide pairs, with each contribution being of only two types, “matched” or “mismatched.” This approximation, although not capturing the molecular interactions in atomistic detail, can represent many salient aspects that have been ignored in previous work on speciation theory. In particular, such a model allows us to include the effects of drift–selection balance in the weak mutation regime ($n\mu_{0}N\ll 1$), due to some phenotypes being coded by more sequences than others and the corresponding effect of population size on speciation dynamics. Recent work has shown that such mappings from genotype to phenotype give rise to a number of nontrivial effects (Force *et al.* 1999; Fontana 2002; Berg *et al.* 2004; Mustonen and Lässig 2005; Khatri *et al.* 2009; Goldstein 2011). Here, we find this simple genotype–phenotype map predicts an increasing rate of accumulating DMIs for decreasing population sizes in the weak mutation regime, the appropriate limit for monomorphically evolving traits, with a robust mechanism that does not require valley crossing by either of the divergent populations. This dependence on population size arises due to two separate mechanisms. First, at large population sizes, the overall substitution rate slows down as the number of nearly neutral mutations decreases, which is line with expectations from the nearly neutral theory (Ohta 1973, 1992). More significantly, the particular form of drift–selection balance that arises from the genotype–phenotype map results in sequence pairs that have a distribution of binding affinities peaked away from the optimal in smaller populations. As a result, less allopatric evolution is required before the hybrid organisms become inviable.

## Materials and Methods

### Quaternary model of transcription factor–DNA binding

Proteins bind DNA through a number of interactions, including electrostatic, van der Waals, and hydrogen bonding at the protein–DNA interface. We can split these interactions into a nonspecific part due mainly to the electrostatic interaction between positive protein side chains and the negative phosphate backbone and a specific part largely due to hydrogen bonding. It is these specific interactions that give rise to discrimination of TFs to different DNA sequences; a TF at its correct sequence binds through both nonspecific and specific interactions, while at a noncorrect site it binds only nonspecifically with an altered conformation that maximizes electrostatic interactions (Von Hippel and Berg 1986).

The two-state approximation (Von Hippel and Berg 1986; Gerland *et al.* 2002) for transcription factors binding at their correct binding sites assumes that amino acid nucleotide interactions are either optimal or nonoptimal and the contribution of each amino acid–nucleotide pair to the total binding energy is approximately additive. The rationale for this model is the underlying biophysics of protein–DNA interactions, in particular, the fact that an amino acid at a protein–DNA interface will tend to have a preferred nucleotide with which to hydrogen bond, taking account of the approximately fixed orientation of the amino acid as positioned by the rest of the protein. The other nucleotides tend to be nonoptimal and not able to hydrogen bond (Takeda *et al.* 1989). Although each optimal interaction is marginally stabilizing [−0.5 kcal/mol (Von Hippel and Berg 1986)], it is the nonoptimal nucleotides that dominate the binding free energy, since the hydrogen bond acceptors and donors in the DNA can neither hydrogen bond to an amino acid nor hydrogen bond to water molecules. This suggests a large cost for each nonoptimal interaction, although in reality the exact value is highly dependent on the particular protein and DNA sequence; empirically measured costs of free energy per amino acid nucleotide mismatch can range from 1–2 kcal/mol (2–3 $k_{B}T$) (Takeda *et al.* 1989; Stormo and Fields 1998) to 4–5 kcal/mol (6–8 $k_{B}T$) (Von Hippel and Berg 1986; Lesser *et al.* 1990; Baldwin 2003), where $k_{B}$ is Boltzmann’s constant and *T* is room temperature. This variation is likely explained by specific cooperative effects that include electrostatic, steric, and solvent interactions (Lesser *et al.* 1990; Baldwin 2003) that change the energy scale of binding dependent on a particular protein–DNA binding context. In this article, for simplicity, we assume a binding energy difference of each nonoptimal interaction compared to an optimal interaction of Δε= 1.8 kcal/mol $=3k_{B}T.$

As mentioned, for each amino acid there tends to be a single nucleotide it prefers to hydrogen bond (Takeda *et al.* 1989). If we designate the category of amino acids by its preferred partnering base (*e.g.*, an amino acid in group T would interact preferably with a thymine) and recognize that only changes of amino acid group affect the binding properties, we can use A, T, C, and G to represent letters from the quaternary alphabet for both proteins and DNA sequences; for simplicity, this assumes that the amino acids are equally distributed among the four categories. In this way, the genome corresponding to this TF–TFBS pair consists of two “genes” of length ℓ in the standard four-letter alphabet of DNA. For simplicity, we consider the mutation rates between amino acid clusters in the protein and nucleotides in the DNA as approximately equal; since our model assumes amino acids and nucleotides are drawn from the same alphabet and as we see below, we treat protein and DNA sequences equally in determining binding affinity, we find in our results that the substitution rates of protein and DNA loci are equal. However, in nature, the rate of substitution between amino acid categories is different from that between DNA bases, increased by the triplet code and decreased by the clustering and other forms of selection acting on the protein, as well as by pleiotropic constraints. However, our model is reasonable, since we would expect the overall dynamics of divergence to be dominated by the loci with the slowest substitution rate and hence slowest effective mutation rate.

Assuming additivity of each amino acid–DNA interaction, the binding free energy will then be equal to a sum of free energies due to matches and mismatches. The number of mismatches is given by the Hamming distance $r=d_{H}\left(g^{P},g^{D}\right),$ where the function $d_{H}$ counts the number of positions where the protein sequence $g^{P}$ and DNA sequence $g^{D}$ are not the same. The number of matches is then ℓ−r, giving a binding free energy,

$$\Delta G=\ell \varepsilon_{m}+\Delta \varepsilon r,$$ (1)

where $\varepsilon_{m}$ is the free energy of each match, which includes both specific and nonspecific interactions. If we choose our zero of energy to be the energy of the best binding sequence, $\ell \varepsilon_{m},$ then we can redefine the binding free energy to be

ΔG=Δεr. (2)

This binding free energy corresponds to the specifically bound mode of attachment (which has both specific and nonspecific contributions). In addition to this specific bound mode, an alternative configuration of protein and DNA exists where the interactions are purely electrostatic. The specific binding mode and this alternative nonspecifically bound mode are in thermodynamic competition. The free energy of binding in the electrostatic nonspecific mode is

$$\Delta G_{\mathrm{ns}}=\ell \Delta \varepsilon_{\mathrm{ns}},$$ (3)

where $\Delta \varepsilon_{\mathrm{ns}}$ is the free energy per nucleotide in the nonspecific mode relative to the optimal binder. Thermodynamic studies of Lac repressor binding to DNA suggest that the difference in free energy between the best specific binding and the nonspecific mode of binding is ∼$15\;k_{B}T,$ so as ℓ=10 for the Lac repressor, we find $\Delta \varepsilon_{\mathrm{ns}}\approx 1.5\;k_{B}T$ (Revzin and Von Hippel 1977; Von Hippel and Berg 1986).

### Modeling the evolution of reproductive isolation

The relationship between the binding energy of a TF to its binding site and the fitness of an organism is poorly understood and is likely very complicated and different for each TF–TFBS pair. There is competition between specific binding and nonspecific binding of the TF (purely electrostatic mode, discussed above). We would expect the fraction of time spent in the specific mode to reach a maximum when there are no mismatches, decreasing with increasing *r* until the TFBS cannot compete with the electrostatic nonspecific mode of binding. Genome-wide studies of TFs in *Escherichia coli* (Mustonen and Lässig 2005) and yeast (Mustonen *et al.* 2008; Haldane *et al.* 2014) found a distribution of binding energies for different TFs that deviated from the random/neutral expectation (Equation 6) for the highest-affinity binders. This deviation from the neutral distribution, which reflects selection for functional binding sites, has a form suggesting a Malthusian fitness landscape that is peaked at nearly optimal binding, decreasing with negative curvature as the binding strength is reduced. These factors suggest a simple model for the fitness landscape where the Malthusian fitness decreases quadratically with the specific binding energy (corresponding to a Gaussian Wrightian fitness function) until a critical number of mismatches r * is reached, corresponding to $\Delta G\left(r\;*\right)=\Delta G_{\mathrm{ns}},$ where nonspecific binding begins to dominate. Beyond this point we consider the organism inviable with a Malthusian fitness of negative infinity (Wrightian fitness of zero). In particular, this cutoff allows us to define DMIs as occurring when r>r * for hybrids between allopatric populations.

More formally,

$$F\left(\Delta G\left(r\right)\right)=\{\begin{array}{cc} -\frac{1}{2}\kappa_{F}r^{2} & \text{for}\;r\leq r\;*\\ -\infty & \text{for}\;r>r*, \end{array}$$ (4)

where $\kappa_{F}$ is the curvature of the fitness landscape and biologically, roughly corresponds to the strength of selection of this trait; as $\kappa_{F}$ decreases the fitness landscape becomes more shallow, and so for a fixed effective population size the landscape becomes more neutral.

Combining $\Delta G\left(r\;*\right)=\Delta G_{\mathrm{ns}}$ with Equations 2 and 3 yields $r*=\ell \Delta \varepsilon_{\mathrm{ns}}/\Delta \varepsilon .$ Note that as binding sites increase in length, ℓ, the stability of the best binder (r=0) relative to nonspecific binding will increase in proportion to ℓ and hence a larger number of mismatches will be required before a binding site becomes nonfunctional. Specifically, for $\Delta \varepsilon =3k_{B}T$ and $\Delta \varepsilon_{ns}=1.5k_{B}T$ (Von Hippel and Berg 1986), we find r*=ℓ/2. In the case of short DNA recognition sites for *Eco*RI endonuclease cleaving DNA, where ℓ=5, it was found that r*≈3 (Lesser *et al.* 1990), which agrees well with our approximate relation between r * and ℓ. We expect our qualitative results to be robust to the choice of such a threshold. Similarly, a more detailed consideration would include binding of the TF to other spurious sites in the genome with large sequence similarity; again we expect such consideration will change the value of ΔG*, but not change the scaling relation r*∝ℓ, as longer binding sites will always have a larger maximum affinity.

To simulate the evolution of TF–TFBS sequence evolution we assume a diploid Wright–Fisher population genetic process with $2N_{e}$ copies of each gene in the population with a fixed effective population size of $N_{e},$ where we have assumed equality with the actual population size *N*. As we are interested in the weak mutation regime ($n\mu_{0}N_{e}\ll 1$), the population is represented by a single fixed sequence for the TF–TFBS pair of loci at each time point, where all individuals are homozygous and mutations are either instantly fixed or eliminated. We use the Gillespie algorithm (Gillespie 1976) to simulate evolution as a continuous-time Markov process; at each step of the simulation the rates of fixation of all 3×2ℓ one-step mutations from the currently fixed alleles (wild type) on both TF and TFBS loci are calculated, and one of these mutations is selected randomly in proportion to the relative rate. Time is then progressed by $K^{-1}\ln\left(u\right),$ where *K* is the sum of the rates of all one-step mutants and *u* is a random number drawn independently between 0 and 1, which ensures the times at which substitutions occur are Poisson distributed, as would be expected for a random substitution process. The rates are based upon the Kimura probability of fixation (Kimura 1962),

$$k=2\mu_{0}N_{e}\frac{1-e^{-2\delta F}}{1-e^{-4N_{e}\delta F}}\approx \mu_{0}\frac{4N_{e}\delta F}{1-e^{-4N_{e}\delta F}},$$ (5)

where δF is the change of fitness of a mutation at a particular location and $2\mu_{0}N_{e}$ is the rate at which mutations arise for each amino acid or nucleotide position in a diploid population; the latter approximation in Equation 5 assumes δF≪1. Note that although in the simulations we use the full form for the fixation probability, fitness effects are typically small (δF≪1) in the simulations, so the substitution rates depend only on the population-scaled fitness changes $4N_{e}\delta F,$ which, for a given mutation, are proportional to $4N_{e}\kappa_{F}.$ In the rest of this article we refer to the scaled population size $4N_{e}\kappa_{F}$ to make it clear that reducing either $N_{e}$ or $\kappa_{F}$ (or both) can change the evolutionary outcomes from those dominated by selection to those dominated by drift.

**Figure 1:**
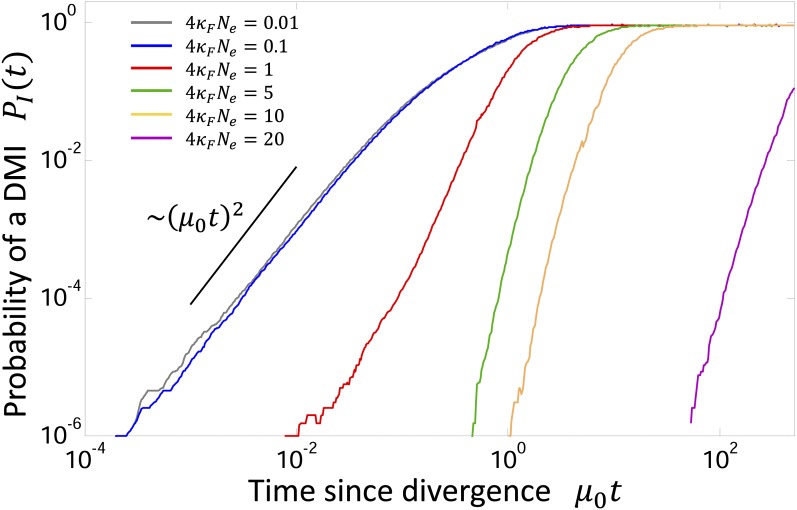
Average probability of a DMI as a function of time after divergence from common ancestor $\mu_{0}t$ calculated from simulations for various scaled population sizes, for ℓ=10.

Using the above evolutionary process based on the biophysics of a TF binding DNA, we study allopatric speciation by independently evolving two lineages in the fitness landscape defined by Equation 4. We create an ancestral genome containing a protein and a DNA binding-site gene, each of length ℓ, with ΔG drawn from the equilibrium distribution of binding energies (Equation 7). This ancestral genome is then duplicated, with each copy representing the start of a different isolated population that subsequently evolves independently. As the allopatric populations evolve, we consider the viability of hybrid offspring of the two lineages. If the evolving protein and DNA sequences in one lineage are $g_{1}^{P}$ and $g_{1}^{D}$ and the other ones are $g_{2}^{P}$ and $g_{2}^{D},$ we can at each time point calculate the Hamming distance for each hybrid as $h_{12}=d_{H}\left(g_{1}^{P},g_{2}^{D}\right)$ and $h_{21}=d_{H}\left(g_{2}^{P},g_{1}^{D}\right)$ with corresponding hybrid binding energies, $\Delta G_{12}^{H}=\Delta \varepsilon h_{12}$ and $\Delta G_{21}^{H}=\Delta \varepsilon h_{21}.$ Using the same fitness function as in Equation 4, we can then evaluate the fitness of the hybrids as a function of time. An incompatibility arises whenever the fitness of the hybrid is −∞ [$h_{12}>r\;*\left(\ell \right)$ or $h_{21}>r\;*\left(\ell \right)$], *i.e.*, when a hybrid TF–TFBS specific binding is weak compared to the nonspecific mode of binding and effectively can no longer recognize its target site. At this point, we assume that the two diverging populations can no longer form viable offspring, and they are reproductively isolated. This is a simplification as hybrid offspring will always be heterozygous at diverged loci, such that the TF–TFBS pair inherited from each parent will have functional binding, while cross-binding between parental pairs will be nonfunctional. Hence, not all postzygotic DMIs would be sufficiently deleterious to affect the viability of these heterozygotic offspring. We assume, however, that there are some TF–TFBS pairs that are sufficiently critical such that r>r * and loss of cross-binding is sufficient to decrease the gene expression level to the extent that the hybrid is inviable; these are the pairs that will be relevant for the speciation process and therefore are the ones addressed by our model. For each scaled population size and sequence length, 1000 replicates were run up to a time of $\mu_{0}t=500,$ allowing us to calculate the probability of the presence of a DMI as a function of divergence time. In addition, simulations were run up to a shorter time (dependent on the exact value of $4\kappa_{F}N_{e}$) with $10^{6}$ replicates to get reliable estimates of the very small probability of a DMI (Figure 1) at early times.

### Data availability

## Results

### Rate of accumulation of hybrid incompatibilities

**Figure 2:**
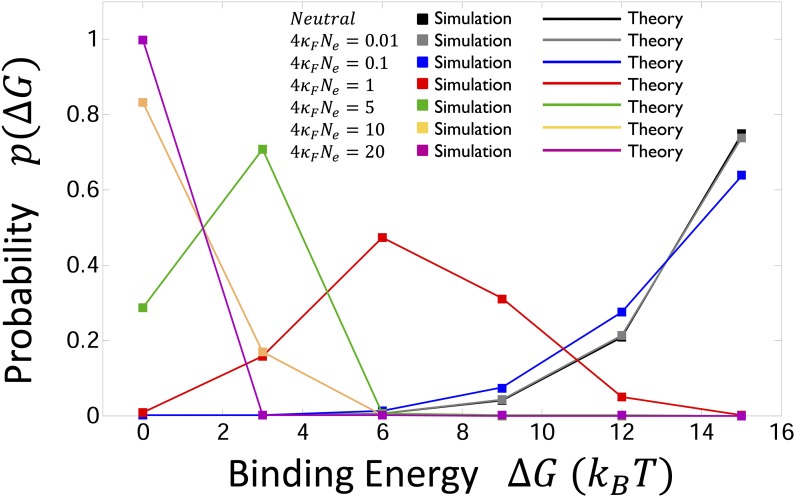
Equilibrium distribution of binding energies ΔG as a result of evolution subject to the quadratic fitness landscape in Equation 4, for ℓ=10. We assume the fitness landscape has a fitness cliff (inviability boundary) for r>r*=ℓ/2=5 mismatches or for binding energies greater than $>\;\Delta \varepsilon r*=15\;k_{B}T,$ which represents when the specific binding energy to its binding site is greater than that of the nonspecific, electrostatic, mode of binding. The solid squares are results of simulations, while the solid lines are the expected distribution from Equation 7, which we see agree very well. In addition, we see that the distribution shifts from one dominated by fitness F(ΔG) at large scaled population sizes ($4\kappa_{F}N\gg 1$) with a peak at the highest fitness binding energy to one dominated by sequence degeneracy at small scaled population sizes ($4\kappa_{F}N\ll 1$), which is peaked at the inviability boundary, representing the left tail of the neutral distribution in Equation 6 (shown in black).

The probability of a DMI $P_{I}\left(t\right)$ as a function of divergence time $\mu_{0}t$ is plotted in Figure 1, for various values of $4\kappa_{F}N_{e}$ for ℓ=10. We see that the model predicts a very strong population size effect for the dynamics of hybrid incompatibilities; as the scaled population size decreases the timescale for DMIs to arise sharply decreases. This effect saturates for very small scaled population sizes, but diverges for very large scaled population sizes, to the point that reproductive isolation will take extremely long times for very large population sizes ($4N_{e}\kappa_{F}\gg 10$). For small scaled population sizes the increase in DMIs is quadratic at small times ($2\ell \mu_{0}t\ll 1$). For large scaled population sizes there is a delayed, but very rapid, increase in DMIs, which does not seem to fit a power law but rather has a negative curvature on a log–log scale. This is consistent with theoretical predictions of a coarse-grained model of TF–DNA binding evolution (Khatri and Goldstein 2015), where the growth of DMIs is rapid with the asymptotic form, as t→0 of $P_{I}\left(t\right)∼\mathrm{erfc}\left(1/\sqrt{t}\right)∼\sqrt{t}e^{-1/t}.$ This form arises when there are nearly neutral diffusive dynamics, as shown by the inset in Supporting Information, Figure S2, and when the common ancestor distribution is very narrow, as shown in Figure 2, in both cases for simulations at large scaled population sizes. We also performed simulations where the common ancestor sequence was drawn to always have the mean binding energy of the equilibrium distribution (Figure S1) and found the results to be nearly identical; this suggests that the power law behavior seen for small populations is not due to averaging over the common ancestor distribution, but as argued in the *Discussion* due to Poissonian distribution of times for substitutions.

The dependence of $P_{I}\left(t\right)$ on population size arises from two effects, the first resulting from the dependence of equilibrium binding strengths on population size. Figure 2 shows the distribution of binding energies on each lineage for different scaled population sizes ($4N_{e}\kappa_{F}$) for ℓ=10 and r*=ℓ/2=5. The distributions are confined to the region 0≤ΔG≤ΔG*, where $\Delta G*=\Delta \varepsilon r*=15\;k_{B}T$ is the inviability boundary. For large scaled population sizes, we see that distributions are peaked near the optimal binding strength ΔG=0, reflecting the efficacy of selection in large populations. However, as the scaled population size is decreased, we see the distribution of binding energies shifts to weaker affinity values (higher ΔG), due to the stronger influence of genetic drift. At the smallest scaled population sizes, genetic drift dominates and the distribution of binding affinities becomes identical to the distribution obtained under neutral evolution (maintaining, however, the inviability boundary). At the level of sequences or genotypes, the neutral distribution is evenly distributed among all possible genotypes; each sequence has equal probability. However, the probability of a given value of ΔG is obtained by multiplying the probability of each sequence times the degeneracy, that is, the number of sequences corresponding to this ΔG. As each sequence has the same probability, the neutral distribution is then simply proportional to the number of sequences that give ΔG or Hamming distance r=ΔG/Δε, which is given by the binomial distribution

$$\Omega \left(\Delta G\left(r\right)\right)=4^{2\ell }\left(\begin{array}{c} \ell \\ r \end{array}\right){\left(\frac{3}{4}\right)}^{r}{\left(\frac{1}{4}\right)}^{\ell -r}.$$ (6)

For example, the number of sequences that give ΔG=0 is $\Omega \left(\Delta G=0\right)=4^{\ell }\approx 10^{6}$ (for ℓ=10), as there is exactly one DNA sequence that matches to each one of the $4^{\ell }$ protein sequences. This number is very small compared to the number of sequences at the inviability border that have five mismatches, $\Omega \left(\Delta G=15\;k_{B}T\right)\approx 6.4\times 10^{10}.$

At intermediate population sizes we can quantify the interplay between selection and degeneracy through the concept of sequence entropy (Barton and Coe 2009; Khatri and Goldstein 2015), representing the (log) number of sequences encoding a given phenotypic state (*e.g.*, binding energy), S(ΔG)=ln(Ω(ΔG)), which is closely related to the Boltzmann entropy from statistical mechanics (Reif 1965). This entropy measure should be distinguished from entropies of sequences due to polymorphisms in the population (in this article we have assumed populations are always monomorphic). The combination of fitness and sequence entropy that is maximized during evolution is the function $\Phi \left(\Delta G\right)=F\left(\Delta G\right)+S\left(\Delta G\right)/4N_{e},$ termed the free fitness (Iwasa 1988; Sella and Hirsh 2005; Haldane *et al.* 2014; Khatri and Goldstein 2015), from which the probability density is given by

$$p\left(\Delta G\right)=\frac{1}{Z}e^{4N_{e}\Phi \left(\Delta G\right)},$$ (7)

where *Z* is a normalization factor, known as the partition function, given by $Z=\sum_{r=0}^{\ell }e^{4N_{e}\Phi \left(\Delta G\right)}.$ This probability density is plotted as solid lines in Figure 2 for different population sizes, using Equations 4, 6, and 7; we see that the agreement between the two is excellent.

The binding energy distributions show that for a general genotype–phenotype map fitness is not maximized, but instead there is a balance between selection for higher fitness and the tendency to undergo drift toward those phenotypes that correspond to the largest number of sequences. As the scaled population size decreases, the initial binding affinity of the common ancestor is on average smaller and so fewer substitutions are required between a pair of divergent lineages for an incompatibility to arise in a hybrid.

**Figure 3:**
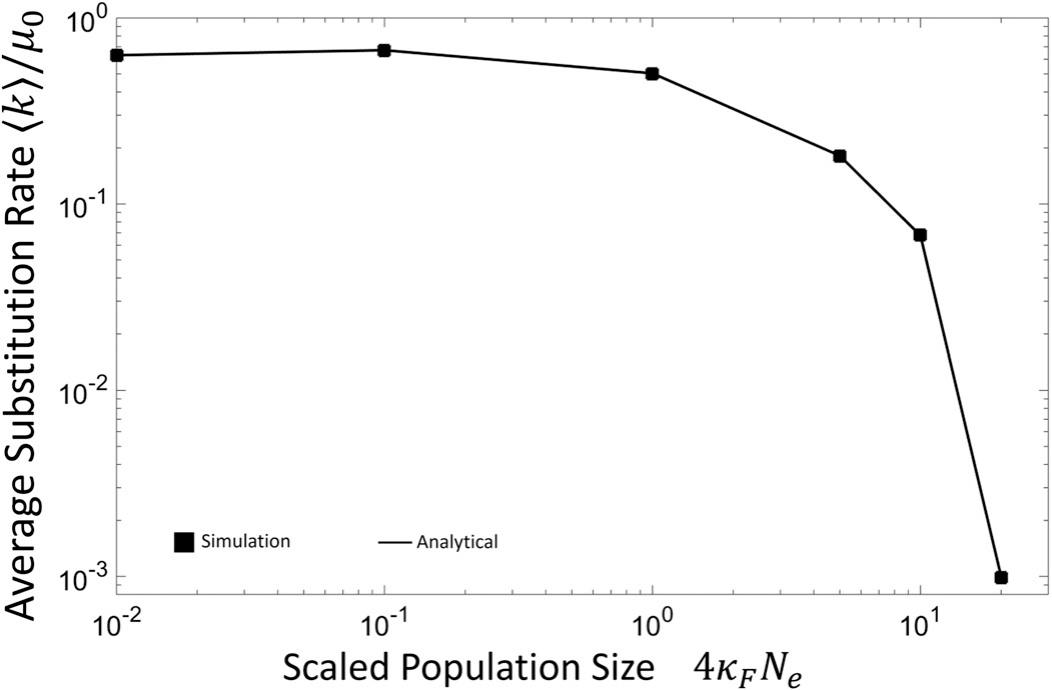
Average total substitution rate for both protein and DNA loci, on a single lineage as function of scaled population size $4\kappa_{F}N.$ Substitution rate is plotted in units of the nucleotide mutation rate $\mu_{0}.$ The solid squares represent simulations, while the solid lines are the theoretical prediction of the average rate $\langle k\rangle =\left(2N_{e}\mu_{0}/3\ell \right)\sum_{r=0}^{r*}p_{\ell }\left(r\right)\left(r\left(\pi^{-}\left(r\right)+1/N_{e}\right)+3\left(\ell -r\right)\pi^{+}\left(r\right)\right),$ where $p_{\ell }\left(r\right)$ is the equilibrium distribution of Hamming distances (shown in Figure 2) and $\pi^{-}$ and $\pi^{+}$ are the fixation probabilities for the transitions r→r−1 and r→r+1, respectively.

The second major factor affecting the rate of accumulation of DMIs is the slowing of the substitution rate with population size, as shown in Figure 3. The dependence we see can be explained by the average size of fitness effects as the scaled population size changes, where $\langle k\rangle ∼\sum_{r}^{r*}p_{\ell }\left(r\right)\left(k_{r\to r+1}+k_{r\to r-1}\right)$ is a sum over terms formed by the product of the equilibrium probability $p_{\ell }\left(r\right)$ and the total substitution rate for r→r±1 (the exact formula given in the legend of Figure 3 and plotted as the solid black line); at very large scaled population sizes the $p_{\ell }\left(r\right)$ is peaked at r=0 and so the average substitution rate will be dominated by transitions between r=0 and r=1. Although $p_{\ell }\left(r\right)$ is maximum for r=0, transitions from r=0 to r=1 happen rarely since it requires fixing a mutant with a population-scaled difference in fitness, $4N_{e}\delta F=-2\kappa_{F}N\Delta \varepsilon^{2},$ which is negative and of magnitude >>1, when $4\kappa_{F}N\gg 1;$ this means substitutions will occur significantly slower than neutral. Conversely, the reverse transition from r=1 to r=0 is also rare, despite the fixation probability being large, since the probability $p_{\ell }(r=1)$ is small due to the same large population-scaled difference in fitness [This must be the case as in equilibrium $p_{\ell }\left(r\right)k_{r\to r+1}=p_{\ell }\left(r+1\right)k_{r+1\to r}$ for the probabilities not to change. This requirement is known in physics as “detailed balance.”]. This explains the slowdown of the accumulation of DMIs for large scaled population sizes observed in Figure 1. However, in very small populations, the inverse of the scaled population size is much larger than differences in fitness so we might expect substitutions to occur at the neutral rate ($\langle k\rangle =\mu_{0}$). In fact, we find that it is roughly half the neutral rate ($\langle k\rangle \approx 0.6\mu_{0}$); this is because for $4\kappa_{F}N_{e}\ll 1$ populations spend a large fraction of the time at the inviability boundary r*, so the substitution rate is diminished compared to the expected neutral rate $\mu_{0},$ since a fraction (ℓ−r*)/ℓ=0.5 of mutations at this boundary are inviable and are never accepted in the population.

**Figure 4:**
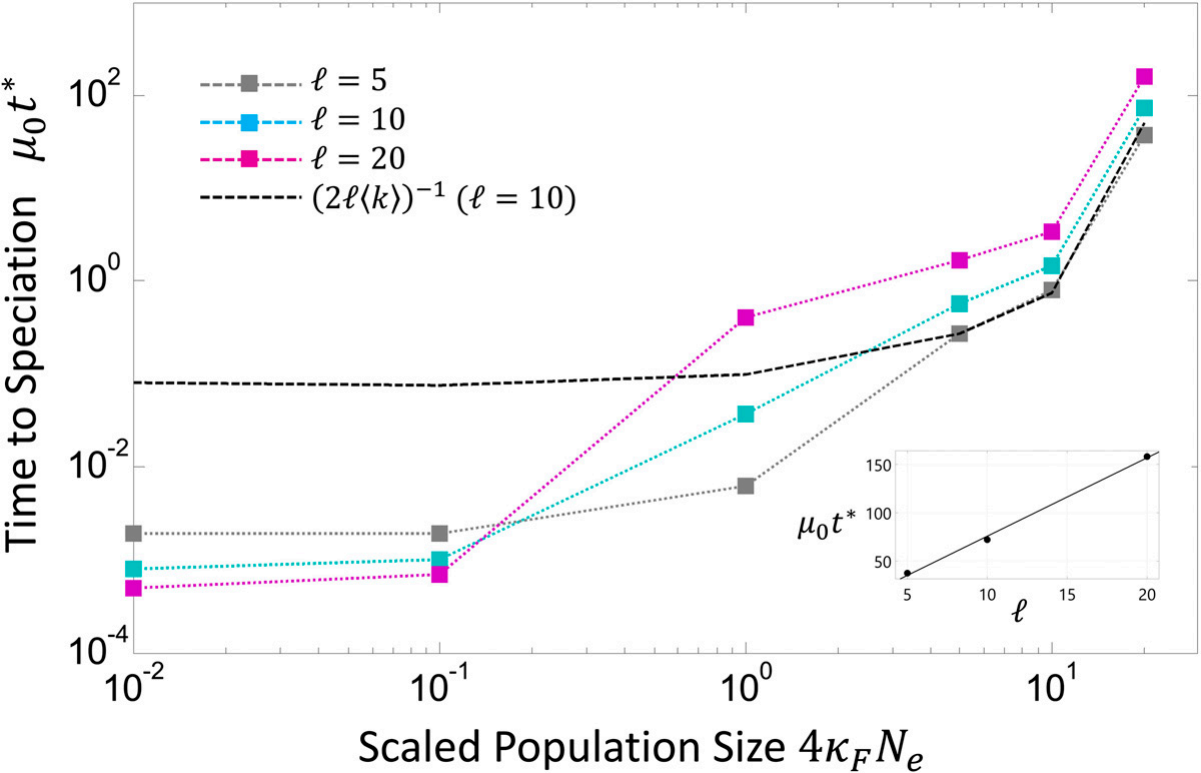
Time for reproductive isolation (RI) to arise as a function of scaled population size $4\kappa_{F}N,$ defined as the time t* when the average probability of a DMI crosses a threshold value of $1/M=10^{-5},$ where *M* is the typical number of interaction partners of a protein in a genome. The black dashed line corresponds to a plot of the inverse of the average substitution rate shown in Figure 3. The inset shows the time to speciation plotted *vs.* sequence length for values of ℓ={5,10,20} (black circles), where the solid line represents the best straight line fit, which indicates that the underlying mechanism of hybrid divergence is neutral diffusion.

Finally, we note that our results are robust with respect to changes in sequence length, showing qualitatively similar behavior for the dynamics of DMIs at different scaled population sizes, as shown in Figure 1. The effect of sequence length is explored in Supporting Information and in Figure 4, which examines the typical time required for RI to arise.

### Estimating the time to reproductive isolation

In a full genome, where there are many possible interacting genes, it will typically be the short-time behavior of each interacting pair that will dominate the development of RI for the whole organism. If we assume ∼m∼10 interaction partners per gene and $n_{G}\approx 2\times 10^{4}$ protein-coding genes, we have ∼$M=\left(1/2\right)mn_{G}\approx 10^{5}$ interaction partners. As only a single one of these interactions giving rise to a DMI is required for RI, we for simplicity estimate the probability that RI has arisen is $P_{\mathrm{RI}}\left(t\right)=1-{\left(1-P_{I}\left(t\right)\right)}^{M},$ which at short times is given by $P_{\mathrm{RI}}\left(t\right)\approx 1-e^{-MP_{I}\left(t\right)}.$ In Figure 4 is plotted the time t * at which $P_{I}\left(t\;*\right)=1/M=10^{-5},$ for ℓ={5,10,20}. We see the rate at which RI develops is strongly dependent on the scaled population size, with a weaker, but still significant dependence on the sequence length. In particular, we see for small scaled populations RI can arise quite quickly, on the timescale of $t*\approx 0.0005/\mu_{0}$ ∼250,000 generations, assuming $\mu_{0}=2\times 10^{-9}.$ There are different aspects of our model, which each cause an underestimate or an overestimate of the time for RI to arise. As discussed, only some fraction of traits will lead to a sufficient change in gene expression to cause an inviable organism, when cross-binding in heterozygotes is eliminated, and so this would cause an underestimate of the time. But on the other hand, particularly for small populations, where the common ancestor binding energy distribution is broad (Figure 2), there will be common ancestor gene pairs, whose binding affinity is closer to the inviability boundary, which would tend to dominate t*, giving a t * that is shorter than our estimate. In addition, not all TF–TFBS pairs will necessarily have optimum fitness at optimum binding, which is likely to cause a reduction of the time to reproductive isolation, as the common ancestor distribution will be peaked closer to the inviability boundary, even in the limit of large populations; this again would mean an overestimation of t *. As discussed above a major determinant at large scaled population sizes of the time for RI to develop is the rate of substitutions on each lineage, the inverse of which is plotted as a dashed line in Figure 4; we see that although the inverse substitution rate is a good predictor for large scaled population sizes, for small scaled populations it fails. This is due to the weaker equilibrium binding affinities at smaller scaled population sizes, which reduces t * further.

The time for RI to arise has a complicated dependence on sequence length, which is explored in detail in Supporting Information. Briefly, for small scaled population sizes ($4\kappa_{F}N_{e}\ll 1$), RI develops more rapidly for longer sequences as the overall substitution or divergence rate ∼ ℓ〈k〉 is roughly proportional to ℓ, yet the distance between the common ancestors and the inviability boundary does not vary appreciably with sequence length. Conversely, for large population sizes ($4\kappa_{F}N_{e}\gg 1$), this trend is reversed and longer sequences develop RI more slowly. This is because, although there is the same dependence of divergence rate on ℓ, the average distance of the common ancestor to the inviability boundary increases linearly with ℓ (r*∝ℓ) due to longer binding sites giving more stable protein–DNA complexes. For large scaled population sizes, as demonstrated in Figure S2 of Supporting Information, the hybrid binding energies have neutral dynamics and so the typical time required to fix r * substitutions will vary quadratically with r * and thus quadratically on ℓ. This quadratic dependence dominates the linear dependence of the divergence rate on ℓ, resulting in an overall linear dependence of t * on ℓ. The speciation times as a function of ℓ for $4\kappa_{F}N_{e}=20$ are shown in the inset in Figure 4; the near-linear dependence lends support to the diffusive model for hybrid dynamics at large population sizes.

## Discussion

Dobzhansky, Muller, and Bateson (Bateson 1909; Dobzhansky 1936; Muller 1942) provided the first solution to Darwin’s conundrum of how speciation might arise by suggesting that in allopatry incompatibilities form between coevolving loci on an epistatic fitness landscape. Here, using a similar approach to that of Tulchinsky *et al.* (2014b), we have examined a biophysically motivated model of how incompatibilities arise in allopatric populations, and their population size dependence, using a simple model of the coevolution of transcription factors binding to DNA in the weak mutation, monomorphic regime. The model of TF–TFBS binding described here is inherently epistatic, despite the assumption that the contribution of each interacting amino acid-nucleotide pair is independent and additive to the total binding energy. Epistasis arises both from the nature of the binding interaction and from the resulting fitnesses. Considering the binding interaction, whether a given amino acid or nucleotide gives rise to a match or mismatch depends on the particular binding partner, so that the binding energy is a nonlinear function of the sequences at the TF and TFBS loci. It is this epistasis that is the source of the Dobzhansky–Muller incompatibilities that we find in our simulations described in *Results*. For example, as has been previously discussed (Johnson and Porter 2000; Tulchinsky *et al.* 2014b) the common ancestor might be fixed for a pair of sequences ATCGC/ATAGC, which has a binding energy of $\Delta G_{\mathrm{CA}}=3k_{B}T,$ as there is only a single mismatch; after a period of divergence, two allopatric populations might be fixed for TTAGC/ATAGC and ATCGA/ATCGC, each arising from just two substitutions, of compensatory effect, from the common ancestor sequence, so that $\Delta G_{1}=\Delta G_{2}=3k_{B}T,$ as there is still only a single mismatch. However, the hybrid sequences are TTAGC/ATCGC and ATCGA/ATAGC, which correspond to binding energies $\Delta G_{12}^{H}=\Delta G_{21}^{H}=6k_{B}T,$ as they each have two mismatches. As the number of substitutions increases on each lineage, we can see that each lineage will maintain good fitness in a stabilizing landscape through compensatory changes, which each try to minimize the number of mismatches; however, each lineage fixes different sets of compensatory mutations, so when combined in a hybrid, the epistasis between pairs of sequences then gives rise to DMIs. The second cause of epistasis is the quadratic dependence of fitness on binding strength, as well as the discontinuity of the fitness function at r=r *. Although there is a similarity between our model and typical polygenic models of quantitative traits, they are very different as for quantitative traits the phenotype is usually modeled as additive in each locus (Wright 1935a,b; Barton 1989), but with quadratic selection inducing epistasis between loci; in our model there is epistasis at the level of phenotype and the fitness of phenotypes.

A key aspect that this biophysical model of evolution introduces to the picture of fitness landscapes is the idea that many sequences can result in the same phenotype. In particular, the number of sequences corresponding to each phenotype can be very different, and this uneven distribution can have important consequences for the evolutionary process. As described, our results arise due to a drift–selection balance, which can be cast in the language of a balance between fitness and sequence entropy. The maximum of the free fitness landscape corresponds to the phenotype when these two evolutionary forces are balanced; importantly, this balance is dependent on the population size. Here, for TF–DNA binding there are many more sequences that have a large number of mismatches compared to those few high-fitness sequences that have a small number of mismatches; at smaller population sizes genetic drift dominates, pushing the equilibrium toward less fit sequences. This has an important consequence for the dynamics of reproductive isolation, that smaller scaled populations on average have common ancestors with a lower equilibrium affinity and so a smaller number of substitutions are needed for a hybrid incompatibility to arise. This leads to the main prediction of this article that smaller scaled populations ($4\kappa_{F}N_{e}\ll 1$) develop incompatibilities more quickly. Note that an evolutionary model that ignored this genotype–phenotype map could not reproduce Figure 2, but would have a common ancestor binding distribution peaked at the best binder for all population sizes, even though the strength of selection is reduced at small scaled population sizes. It should be stressed that the key parameter of interest is the scaled population size and so our results do not apply to just small populations, but in principle to TF–TFBS pairs in organisms of large absolute population size, but weak absolute selection, such that $4\kappa_{F}N\ll 1;$ again across the genome there are likely many pairs of TF–TFBS, for which $4\kappa_{F}N_{e}\ll 1,$ affording the possibility for rapid reproductive isolation to arise under stabilizing selection. For example, human studies suggest that ∼20% of mutations in amino acids are under weak selection, such that $4\kappa_{F}N_{e}\ll 1$ (Eyre-Walker *et al.* 2006), and so some fraction of these would be related to TF–TFBS interactions to which our model would apply. In general, the rate of reproductive isolation due to stabilizing selection will depend on the underlying distribution of fitness effects produced by new mutations in a given organism; if this distribution is assumed roughly fixed independent of the organism, then we would expect the proportion of TF–TFBS pairs that fall into the weak selection category to increase for smaller populations and the average rate of developing RI (per locus pair) will be higher compared to that in larger populations.

At larger scaled population sizes ($4\kappa_{F}N_{e}\gg 1,$ but still in the weak mutation regime, $n\mu_{0}N\ll 1$), where fitness dominates drift we find this trend continues, but for a different reason; when $4\kappa_{F}N_{e}\gg 1,$ populations no longer diverge neutrally and instead need to fix deleterious mutants whose difference in fitness is large compared to the inverse of the effective population size. This means that the time for reproductive isolation becomes very long for very large scaled populations. Overall, this picture is consistent with predictions of the nearly neutral theory, where large populations have a diminishing substitution rate (Ohta 1973, 1992; Lanfear *et al.* 2014). However, while there is evidence consistent with the nearly neutral theory from experimental studies (Wu and Li 1985; Ohta 1995; Johnson and Seger 2001; Weinreich 2001), they are not yet conclusive. In addition, there are theoretical models that predict no dependence on population size of the population-scaled fitness effects (Cherry 1998; Goldstein 2013), depending on the exact nature of the genotype–phenotype map. Again it should be noted that it is the effective scaled population size of the loci of interest that is key and so our model specifically predicts that TF–TFBS pairs in a genome under stabilizing selection and for which $4\kappa_{F}N_{e}\gg 1$ are unlikely to give rise to RI; however, this can in principle occur in large or small absolute populations, depending on the strength of selection on TF–TFBS pairs. Again, assuming a roughly fixed distribution of fitness effects, our results would suggest that for larger populations, the mechanism we describe under stabilizing selection would be relatively unimportant in contributing to RI.

As discussed in the Introduction there is some empirical evidence that smaller populations develop postzygotic isolation more quickly, although there have yet to be any systematic or definite studies. Our model then provides a rationale for these observations in the field with a robust mechanism that does not require that either lineage pass through a fitness valley. It also provides an insight, through a biophysical model, of the mechanistic causes of how DMIs develop for coevolved pairwise molecular interactions. While we would not expect quantitative agreement with biological systems, we can make a rough comparison to empirical data: our results suggest that reproductive isolation can occur on a timescale of the order of a few hundred thousand generations for small scaled population sizes. Direct studies of interspecific hybrids of African cichlids (Stelkens *et al.* 2010) show that postzygotic isolation typically arises over a timescale of ∼ 4 − 18 MY, which corresponds to ∼ 1 − 6 million generations, assuming a generation time of 3 years (Nagl *et al.* 1998), which suggests the mechanism we present is roughly consistent with empirical data. Importantly, we see that this mechanism, which poises small populations at the inviability boundary, can provide relatively rapid reproductive isolation between lineages with only nearly neutral evolution, without having to invoke valley crossing or peak shifts, or positive selection, which requires large populations.

Overall, our results suggest that stabilizing selection via the mechanism studied (and its analogs for more complicated gene regulatory systems) would have more importance at smaller population sizes and less at larger population sizes; this latter assertion is consistent with a number of speciation genes found to show evidence of positive selection, in many cases as a result of genomic conflict (Johnson 2010; Presgraves 2010), which are predominantly in *Drosophila*, which has a large effective population size and for which it is known that positive selection is quite pervasive (Andolfatto and Przeworski 2000; Macpherson *et al.* 2007). However, it is difficult as yet to draw strong general conclusions about the relative role of positive *vs.* stabilizing selection as a cause of DMIs, although this work highlights the relative role that different mechanisms might play at different population sizes and gives a quantitative theory that experimentalists can use to look for direct signatures of RI arising due to stabilizing selection.

The model studied, however, is simplified compared to the complexity of gene regulation in eukaryotes with multiple TFs binding to enhancers to control gene transcription and each TF having multiple binding sites controlling many different genes. Here, we treat TFs and their binding sites on an equal footing and so, for example, the substitution rate in each is the same. It is commonly thought that since TFs are under stronger pleiotropic constraints, they evolve more slowly and so much of the phenotypic divergence between species is driven by *cis*-regulatory change (King and Wilson 1975; Wittkopp *et al.* 2008) (and reviewed recently by Lynch and Wagner 2008). We expect that as pleiotropy will act to reduce the substitution rate on a TF, the divergence rate of allopatric lineages will decrease. This suggests that if pleiotropy is important, our simulations may underestimate the average time to reproductive isolation. However, a similar biophysical model (Tulchinsky *et al.* 2014a), albeit in the strong mutation regime, shows that for the case of a single TF binding two functional binding sites, despite the additional constraint, incompatibilities can arise at similar rates to those of a single TF–TFBS pair under stabilizing selection.

Previous theoretical work by Orr (1995; Orr and Turelli 2001) predicts that in the weak mutation regime, the number of incompatibilities should increase as $∼\;t^{2}$ from a fixed common ancestor, due to the combinatorial possibilities over a large number of pairwise interacting loci. Here, we predict the same growth of DMIs with time, but only for small scaled population sizes ($4\kappa_{F}N\ll 1$) and for a single two-locus system. However, the underlying mechanism appears to be very different here and not likely to be universal. Simulations with a fixed common ancestor rather than one drawn from the equilibrium distribution (Equation 7) are nearly identical (Figure S1 in Supporting Information); this suggests that the power law arises (here quadratic) mainly due to the close proximity of the common ancestor to the inviability boundary, requiring just a few substitutions in each lineage, and so the number of DMIs at short times is dominated by how likely a few substitutions are to arrive very quickly. This is given by a Poisson distribution, so if K * substitutions are needed on average for an incompatibility, then $P_{I}\left(K*;\mu t\right)={\left(\mu t\right)}^{K*}e^{-\mu t}/K\;*!,$ which for short times μt≪1, $P_{I}\left(K*;\mu t\right)∼{\left(\mu t\right)}^{K*}$ to leading order in μt. This suggests that for a quaternary alphabet K*≈2, which is the minimum number of substitutions required for an incompatibility to arise, since a single substitution in one lineage will always give rise to the common ancestor and mutated genotype in the hybrids. On the other hand, for large populations, which have a peaked distribution of common ancestors relative to the Hamming distance to the inviability threshold r*, we observe that the growth of DMIs does not appear to be described by a simple power law, but instead the results suggest there is a negative curvature to their growth on a log–log plot. In addition, we find that the variance of binding energies increases linearly with time in the limit of large populations (inset in Figure S2 in Supporting Information), so together with our results that indicate t*∼ℓ (inset in Figure 4), this suggests that the hybrid binding energies follow neutral diffusive dynamics for large scaled population sizes. This is as predicted by a simple calculation of the growth of DMIs due to a continuous diffusion model for the evolution of TF–DNA binding (Khatri and Goldstein 2015) and arises at large scaled population sizes due to the fact that from a fixed common ancestor there is a large mutational distance that needs to be diffused by hybrids before incompatibilities can arise. We suggest that more detailed studies of species divergence, similar to current works (Matute *et al.* 2010; Moyle and Nakazato 2010), which show a rapid increase in DMIs, should be able to discern between these two qualitatively different behaviors at different population sizes. In particular, recent cross-species ChiP-seq analysis of transcription factor binding (Schmidt *et al.* 2010) suggests a way to explicitly test our predictions at the level of actual binding affinities of hybrid TF–TFBS combinations for recently diverged species, such as in the *Drosophila* family.

The process of speciation underlies the vast diversity of life on Earth. Gene expression divergence is thought to underlie many differences between species (King and Wilson 1975; Wray 2007; Wolf *et al.* 2010), for example, in the Galapagos finches (Abzhanov *et al.* 2006), in the various species of *Drosophila* (Wittkopp *et al.* 2008), and with more direct evidence of a role in speciation through the evolution of genes related to transcription factors (Ting *et al.* 1998; Brideau *et al.* 2006). More recently studies of crosses between *D. melanogaster* and *D. santomea*, which diverged >10 million years ago, have revealed how the cryptic divergence of genetic architecture of conserved developmental body plans leads to postzygotic isolation (Gavin-Smyth and Matute 2013). Proteins binding to DNA to control gene expression are a prototypical coevolving system and critical for the proper development of organisms; thus these results have strong implications for speciation rates and diversity of populations at small population sizes. In addition, although our model is motivated by DNA–protein binding, the approach could be adapted to any type of interacting macromolecules, for example, coevolution of protein–protein interactions or the interaction of genes expressed by the nucleus and mitochondria, where in particular such interactions have been shown in yeast to give rise to cytonuclear incompatibilities (Chou and Leu 2010; Chou *et al.* 2010).

## Supplementary Material

Supporting Information

## References

[bib1] Abzhanov A. Kuo W. P. Hartmann C. Grant B. R. Grant P. R., 2006 The calmodulin pathway and evolution of elongated beak morphology in Darwin’s finches. Nature 442: 563–567. 16885984 10.1038/nature04843

[bib2] Andolfatto P. Przeworski M., 2000 A genome-wide departure from the standard neutral model in natural populations of *Drosophila*. Genetics 156: 257–268. 10978290 10.1093/genetics/156.1.257 PMC1461228

[bib3] Ayala F. J. Campbell C. D. Selander R. K., 1996 Molecular population genetics of the alcohol dehydrogenase locus in the Hawaiian drosophilid D. mimica. Mol. Biol. Evol. 13: 1363–1367. 8952080 10.1093/oxfordjournals.molbev.a025582

[bib4] Baldwin R. L., 2003 In search of the energetic role of peptide hydrogen bonds. J. Biol. Chem. 278: 17581–17588. 12582164 10.1074/jbc.X200009200

[bib5] Barraclough T. G. Nee S., 2001 Phylogenetics and speciation. Trends Ecol. Evol. 16: 391–399. 11403872 10.1016/s0169-5347(01)02161-9

[bib6] Barton N., 1989 The divergence of a polygenic system subject to stabilizing selection, mutation and drift. Genet. Res. 54: 59–77. 2806907 10.1017/s0016672300028378

[bib7] Barton N. Rouhani S., 1987 The frequency of shifts between alternative equilibria. J. Theor. Biol. 125: 397–418. 3657219 10.1016/s0022-5193(87)80210-2

[bib8] Barton N. H. Charlesworth B., 1984 Genetic revolutions, founder effects, and speciation. Annu. Rev. Ecol. Syst. 15: 133–164.

[bib9] Barton N. H. Coe J. B., 2009 On the application of statistical physics to evolutionary biology. J. Theor. Biol. 259: 317–324. 19348811 10.1016/j.jtbi.2009.03.019

[bib10] Bateson W., 1909 Darwin and Modern Science. Cambridge University Press, New York, pp. 85–101.

[bib11] Berg J. Willmann S. Lässig M., 2004 Adaptive evolution of transcription factor binding sites. BMC Evol. Biol. 4: 42. 15511291 10.1186/1471-2148-4-42 PMC535555

[bib12] Brideau, N. J., H. A. Flores, J. Wang, S. Maheshwari, X. Wang *et al*., 2006 Two Dobzhansky-Muller genes interact to cause hybrid lethality in Drosophila. Science 314: 1292–1295. 10.1126/science.1133953 17124320

[bib13] Cherry J. L., 1998 Should we expect substitution rate to depend on population size? Genetics 150: 911–919. 9755219 10.1093/genetics/150.2.911 PMC1460373

[bib14] Chou J.-Y. Leu J.-Y., 2010 Speciation through cytonuclear incompatibility: insights from yeast and implications for higher eukaryotes. BioEssays 32: 401–411. 20414898 10.1002/bies.200900162

[bib15] Chou J.-Y. Hung Y.-S. Lin K.-H. Lee H.-Y. Leu J.-Y., 2010 Multiple molecular mechanisms cause reproductive isolation between three yeast species. PLoS Biol. 8: e1000432. 20652018 10.1371/journal.pbio.1000432 PMC2907292

[bib16] Cooper A. Penny D., 1997 Mass survival of birds across the cretaceous-tertiary boundary: molecular evidence. Science 275: 1109–1113. 9027308 10.1126/science.275.5303.1109

[bib17] Coyne, J. A., and H. A. Orr, 2004 *Speciation*. Sinauer Associates, Sunderland, MA.

[bib18] Dobzhansky T., 1936 Studies on hybrid sterility. ii. Localization of sterility factors in *Drosophila pseudoobscura* hybrids. Genetics 21: 113–135. 17246786 10.1093/genetics/21.2.113 PMC1208664

[bib19] Eyre-Walker A. Woolfit M. Phelps T., 2006 The distribution of fitness effects of new deleterious amino acid mutations in humans. Genetics 173: 891–900. 16547091 10.1534/genetics.106.057570 PMC1526495

[bib20] Fitzpatrick B. M., 2004 Rates of evolution of hybrid inviability in birds and mammals. Evolution 58: 1865–1870. 15446440 10.1111/j.0014-3820.2004.tb00471.x

[bib21] Fiumera A. Parker P. Fuerst P., 2000 Effective population size and maintenance of genetic diversity in captive-bred populations of a Lake Victoria cichlid. Conserv. Biol. 14: 886–892.

[bib22] Fontana W., 2002 Modelling ‘evo-devo’ with RNA. BioEssays 24: 1164–1177. 12447981 10.1002/bies.10190

[bib23] Force A. Lynch M. Pickett F. B. Amores A. Yan Y. L., 1999 Preservation of duplicate genes by complementary, degenerative mutations. Genetics 151: 1531–1545. 10101175 10.1093/genetics/151.4.1531 PMC1460548

[bib24] Gavin-Smyth J. Matute D. R., 2013 Embryonic lethality leads to hybrid male inviability in hybrids between Drosophila melanogaster and D. santomea. Ecol. Evol. 3: 1580–1589. 23789069 10.1002/ece3.573 PMC3686193

[bib25] Gavrilets S., 1999 A dynamical theory of speciation on holey adaptive landscapes. Am. Nat. 154: 1–22. 10.1086/303217 29587497

[bib26] Gavrilets S., 2003 Perspective: models of speciation: What have we learned in 40 years? Evolution 57: 2197–2215. 14628909 10.1111/j.0014-3820.2003.tb00233.x

[bib27] Gavrilets, S., 2004 *Fitness Landscapes and the Origin of Species*. Princeton University Press, Princeton, NJ.

[bib28] Gerland U. Moroz J. D. Hwa T., 2002 Physical constraints and functional characteristics of transcription factor-DNA interaction. Proc. Natl. Acad. Sci. USA 99: 12015–12020. 12218191 10.1073/pnas.192693599 PMC129390

[bib29] Gillespie D. T., 1976 A general method for numerically simulating the stochastic time evolution of coupled chemical reactions. J. Comput. Phys. 22: 403–434.

[bib30] Glor R. E. Gifford M. E. Larson A. Losos J. B. Schettino L. R., 2004 Partial island submergence and speciation in an adaptive radiation: a multilocus analysis of the Cuban green anoles. Proc. R. Soc. Lond. B Biol. Sci. 271: 2257–2265. 10.1098/rspb.2004.2819 PMC1691862 15539351

[bib31] Goldstein R. A., 2011 The evolution and evolutionary consequences of marginal thermostability in proteins. Proteins 79: 1396–1407. 21337623 10.1002/prot.22964

[bib32] Goldstein R. A., 2013 Population size dependence of fitness effect distribution and substitution rate probed by biophysical model of protein thermostability. Genome Biol. Evol. 5: 1584–1593. 23884461 10.1093/gbe/evt110 PMC3787666

[bib33] Haldane A. Manhart M. Morozov A. V., 2014 Biophysical fitness landscapes for transcription factor binding sites. PLoS Comput. Biol. 10: e1003683. 25010228 10.1371/journal.pcbi.1003683 PMC4091707

[bib34] Iwasa Y., 1988 Free fitness that always increases in evolution. J. Theor. Biol. 135: 265–281. 3256719 10.1016/s0022-5193(88)80243-1

[bib35] Johnson K. P. Seger J., 2001 Elevated rates of nonsynonymous substitution in island birds. Mol. Biol. Evol. 18: 874–881. 11319271 10.1093/oxfordjournals.molbev.a003869

[bib36] Johnson N. A., 2010 Hybrid incompatibility genes: remnants of a genomic battlefield? Trends Genet. 26: 317–325. 20621759 10.1016/j.tig.2010.04.005

[bib37] Johnson N. A. Porter A. H., 2000 Rapid speciation via parallel, directional selection on regulatory genetic pathways. J. Theor. Biol. 205: 527–542. 10931750 10.1006/jtbi.2000.2070

[bib38] Johnson N. A. Porter A. H., 2007 Evolution of branched regulatory genetic pathways: directional selection on pleiotropic loci accelerates developmental system drift. Genetica 129: 57–70. 16912839 10.1007/s10709-006-0033-2

[bib39] Khatri B. S. Goldstein R. A., 2015 A coarse-grained biophysical model of sequence evolution and the population size dependence of the speciation rate. J. Theor. Biol. 378: 56–64. 25936759 10.1016/j.jtbi.2015.04.027 PMC4457359

[bib40] Khatri B. S. McLeish T. C. B. Sear R. P., 2009 Statistical mechanics of convergent evolution in spatial patterning. Proc. Natl. Acad. Sci. USA 106: 9564–9569. 19497876 10.1073/pnas.0812260106 PMC2701012

[bib41] Kimura M., 1962 On the probability of fixation of mutant genes in a population. Genetics 47: 713–719. 14456043 10.1093/genetics/47.6.713 PMC1210364

[bib42] King M. C. Wilson A. C., 1975 Evolution at two levels in humans and chimpanzees. Science 188: 107–116. 1090005 10.1126/science.1090005

[bib43] Lande R., 1979 Effective deme sizes during long-term evolution estimated from rates of chromosomal rearrangement. Evolution 33: 234–251. 10.1111/j.1558-5646.1979.tb04678.x 28568063

[bib44] Lande R., 1985 Expected time for random genetic drift of a population between stable phenotypic states. Proc. Natl. Acad. Sci. USA 82: 7641–7645. 3865184 10.1073/pnas.82.22.7641 PMC391389

[bib45] Landry C. R. Wittkopp P. J. Taubes C. H. Ranz J. M. Clark A. G., 2005 Compensatory *cis-trans* evolution and the dysregulation of gene expression in interspecific hybrids of *Drosophila*. Genetics 171: 1813–1822. 16143608 10.1534/genetics.105.047449 PMC1456106

[bib46] Lanfear R. Kokko H. Eyre-Walker A., 2014 Population size and the rate of evolution. Trends Ecol. Evol. 29: 33–41. 24148292 10.1016/j.tree.2013.09.009

[bib47] Lesser D. R. Kurpiewski M. R. Jen-Jacobson L., 1990 The energetic basis of specificity in the Eco RI endonuclease–DNA interaction. Science 250: 776–786. 2237428 10.1126/science.2237428

[bib48] Lynch V. J. Wagner G. P., 2008 Resurrecting the role of transcription factor change in developmental evolution. Evolution 62: 2131–2154. 18564379 10.1111/j.1558-5646.2008.00440.x

[bib49] Macpherson J. M. Sella G. Davis J. C. Petrov D. A., 2007 Genomewide spatial correspondence between nonsynonymous divergence and neutral polymorphism reveals extensive adaptation in *Drosophila*. Genetics 177: 2083–2099. 18073425 10.1534/genetics.107.080226 PMC2219485

[bib50] Matute D. R. Butler I. A. Turissini D. A. Coyne J. A., 2010 A test of the snowball theory for the rate of evolution of hybrid incompatibilities. Science 329: 1518–1521. 20847270 10.1126/science.1193440

[bib51] Mayr E., 1954 Geographic speciation in tropical echinoids. Evolution 8: 1–18.

[bib52] Mayr, E., 1970 *Populations*, *Species*, *and Evolution*. Harvard University Press, Cambridge, MA, pp. 347–350.

[bib53] Moyle L. C. Nakazato T., 2010 Hybrid incompatibility “snowballs” between solanum species. Science 329: 1521–1523. 20847271 10.1126/science.1193063

[bib54] Muller, H., 1942 Isolating mechanisms, evolution and temperature. Biol. Symp. 6: 71–125.

[bib55] Mustonen V. Lässig M., 2005 Evolutionary population genetics of promoters: predicting binding sites and functional phylogenies. Proc. Natl. Acad. Sci. USA 102: 15936–15941. 16236723 10.1073/pnas.0505537102 PMC1276062

[bib56] Mustonen V. Kinney J. Callan C. G. Lässig M., 2008 Energy-dependent fitness: a quantitative model for the evolution of yeast transcription factor binding sites. Proc. Natl. Acad. Sci. USA 105: 12376–12381. 18723669 10.1073/pnas.0805909105 PMC2527919

[bib57] Nagl S. Tichy H. Mayer W. E. Takahata N. Klein J., 1998 Persistence of neutral polymorphisms in Lake Victoria cichlid fish. Proc. Natl. Acad. Sci. USA 95: 14238–14243. 9826684 10.1073/pnas.95.24.14238 PMC24357

[bib58] Nee S., 2001 Inferring speciation rates from phylogenies. Evolution 55: 661–668. 11392383 10.1554/0014-3820(2001)055[0661:isrfp]2.0.co;2

[bib59] Nei M. Maruyama T. Wu C. I., 1983 Models of evolution of reproductive isolation. Genetics 103: 557–579. 6840540 10.1093/genetics/103.3.557 PMC1202040

[bib60] Ohta T., 1973 Slightly deleterious mutant substitutions in evolution. Nature 246: 96–98. 4585855 10.1038/246096a0

[bib61] Ohta T., 1992 The nearly neutral theory of molecular evolution. Annu. Rev. Ecol. Syst. 23: 263–286.

[bib62] Ohta T., 1995 Synonymous and nonsynonymous substitutions in mammalian genes and the nearly neutral theory. J. Mol. Evol. 40: 56–63. 7714912 10.1007/BF00166595

[bib63] Oppen M. Turner G. Rico C. Deutsch J. Ibrahim K., 1997 Unusually fine-scale genetic structuring found in rapidly speciating Malawi cichlid fishes. Proc. Biol. Sci. 264: 1803–1812.

[bib64] Orr H. Orr L., 1996 Waiting for speciation: the effect of population subdivision on the time to speciation. Evolution 50: 1742–1749. 10.1111/j.1558-5646.1996.tb03561.x 28565607

[bib65] Orr H. A., 1995 The population genetics of speciation: the evolution of hybrid incompatibilities. Genetics 139: 1805–1813. 7789779 10.1093/genetics/139.4.1805 PMC1206504

[bib66] Orr H. A., 2001 The genetics of species differences. Trends Ecol. Evol. 16: 343–350. 11403866 10.1016/s0169-5347(01)02167-x

[bib67] Orr H. A. Turelli M., 2001 The evolution of postzygotic isolation: accumulating Dobzhansky-Muller incompatibilities. Evolution 55: 1085–1094. 11475044 10.1111/j.0014-3820.2001.tb00628.x

[bib68] Owen R. Crossley R. Johnson T. Tweddle D. Kornfield I., 1990 Major low levels of Lake Malawi and their implications for speciation rates in cichlid fishes. Proc. R. Soc. Lond. B Biol. Sci. 240: 519–553.

[bib69] Presgraves D. C., 2010 The molecular evolutionary basis of species formation. Nat. Rev. Genet. 11: 175–180. 20051985 10.1038/nrg2718

[bib70] Reif, F., 1965 *Fundamentals of Statistical and Thermal Physics*. McGraw-Hill, New York.

[bib71] Revzin A. Von Hippel P. H., 1977 Direct measurement of association constants for the binding of Escherichia coli lac repressor to non-operator DNA. Biochemistry 16: 4769–4776. 20938 10.1021/bi00641a002

[bib72] Rubinoff R. W. Rubinoff I., 1971 Geographic and reproductive isolation in Atlantic and Pacific populations of Panamanian Bathygobius. Evolution 25: 88–97. 10.1111/j.1558-5646.1971.tb01861.x 28562942

[bib73] Santos M. E. Salzburger W., 2012 Evolution. How cichlids diversify. Science 338: 619–621. 23118176 10.1126/science.1224818

[bib74] Sawai H. Kim H. L. Kuno K. Suzuki S. Gotoh H., 2010 The origin and genetic variation of domestic chickens with special reference to junglefowls Gallus g. gallus and G. varius. PLoS One 5: e10639. 20502703 10.1371/journal.pone.0010639 PMC2873279

[bib75] Schmidt D. Wilson M. D. Ballester B. Schwalie P. C. Brown G. D., 2010 Five-vertebrate chip-seq reveals the evolutionary dynamics of transcription factor binding. Science 328: 1036–1040. 20378774 10.1126/science.1186176 PMC3008766

[bib76] Seehausen O. Butlin R. K. Keller I. Wagner C. E. Boughman J. W., 2014 Genomics and the origin of species. Nat. Rev. Genet. 15: 176–192. 24535286 10.1038/nrg3644

[bib77] Sella G. Hirsh A. E., 2005 The application of statistical physics to evolutionary biology. Proc. Natl. Acad. Sci. USA 102: 9541–9546. 15980155 10.1073/pnas.0501865102 PMC1172247

[bib78] Stelkens R. B. Young K. A. Seehausen O., 2010 The accumulation of reproductive incompatibilities in African cichlid fish. Evolution 64: 617–633. 19796149 10.1111/j.1558-5646.2009.00849.x

[bib79] Stormo G. D. Fields D. S., 1998 Specificity, free energy and information content in protein-DNA interactions. Trends Biochem. Sci. 23: 109–113. 9581503 10.1016/s0968-0004(98)01187-6

[bib80] Takeda Y. Sarai A. Rivera V. M., 1989 Analysis of the sequence-specific interactions between cro repressor and operator DNA by systematic base substitution experiments. Proc. Natl. Acad. Sci. USA 86: 439–443. 2911590 10.1073/pnas.86.2.439 PMC286485

[bib81] Ting C.-T. Tsaur S.-C. Wu M.-L. Wu C.-I., 1998 A rapidly evolving homeobox at the site of a hybrid sterility gene. Science 282: 1501–1504. 9822383 10.1126/science.282.5393.1501

[bib82] Tulchinsky A. Y. Johnson N. A. Porter A. H., 2014 a Pleiotropic constraint and compensation in the evolution of hybrid incompatibility in a sequence-based bioenergetic model of transcription factor binding. Genetics 198: 1645–1654. 25313130 10.1534/genetics.114.171397 PMC4256777

[bib83] Tulchinsky A. Y. Johnson N. A. Watt W. B. Porter A. H., 2014 b Hybrid incompatibility arises in a sequence-based bioenergetic model of transcription factor binding. Genetics 198: 1155–1166. 25173845 10.1534/genetics.114.168112 PMC4224158

[bib84] von Hippel P. H. Berg O. G., 1986 On the specificity of DNA-protein interactions. Proc. Natl. Acad. Sci. USA 83: 1608–1612. 3456604 10.1073/pnas.83.6.1608 PMC323132

[bib85] Weinreich D. M., 2001 The rates of molecular evolution in rodent and primate mitochondrial DNA. J. Mol. Evol. 52: 40–50. 11139293 10.1007/s002390010132

[bib86] Wittkopp P. J. Haerum B. K. Clark A. G., 2008 Regulatory changes underlying expression differences within and between Drosophila species. Nat. Genet. 40: 346–350. 18278046 10.1038/ng.77

[bib87] Wolf J. B. Lindell J. Backström N., 2010 Speciation genetics: current status and evolving approaches. Philos. Trans. R. Soc. Lond. B Biol. Sci. 365: 1717–1733. 20439277 10.1098/rstb.2010.0023 PMC2871893

[bib88] Wray G. A., 2007 The evolutionary significance of cis-regulatory mutations. Nat. Rev. Genet. 8: 206–216. 17304246 10.1038/nrg2063

[bib89] Wright S., 1935 a The analysis of variance and the correlations between relatives with respect to deviations from an optimum. J. Genet. 30: 243–256.

[bib90] Wright S., 1935 b Evolution in populations in approximate equilibrium. J. Genet. 30: 257–266.

[bib91] Wu C.-I. Li W.-H., 1985 Evidence for higher rates of nucleotide substitution in rodents than in man. Proc. Natl. Acad. Sci. USA 82: 1741–1745. 3856856 10.1073/pnas.82.6.1741 PMC397348

[bib92] Wu C.-I. Ting C.-T., 2004 Genes and speciation. Nat. Rev. Genet. 5: 114–122. 14735122 10.1038/nrg1269

