# Supplementary material for: Simple Biophysical Model Predicts Faster Accumulation of Hybrid Incompatibilities in Small Populations Under Stabilizing Selection
Source: Genetics. 2015 Oct 3;201(4):1525–37. doi: 10.1534/genetics.115.181685 (PMC4676520; doi:10.1534/genetics.115.181685)
Supplement: Supporting Information [file supp_201_4_1525__index.html]

Simple Biophysical Model Predicts Faster Accumulation of Hybrid Incompatibilities in Small Populations Under Stabilizing Selection — Supporting Information 

# Simple Biophysical Model Predicts Faster Accumulation of Hybrid Incompatibilities in Small Populations Under Stabilizing Selection

**Files in this data supplement:**

- Supporting Information: Figures S1-S4 and Supporting Text (PDF, 258 KB)
