# Supplementary material for: Simple Biophysical Model Predicts Faster Accumulation of Hybrid Incompatibilities in Small Populations Under Stabilizing Selection
Source: Genetics. 2015 Oct 3;201(4):1525–37. doi: 10.1534/genetics.115.181685 (PMC4676520; doi:10.1534/genetics.115.181685)
Supplement: Supporting Information [file 10.1534_115.181685_genetics.115.181685-1.pdf]

Supplementary Material: Simple biophysical model predicts faster accumulation of hybrid incompatibilities in small populations under stabilising selection

Bhavin S. Khatri[*,1] **and Richard A. Goldstein**[†]
[*]The Francis Crick Institute, Mill Hill Laboratory, London, United Kingdom, [†]Division of Infection & Immunity, University College London, London, United Kingdom

Probability of a DMI with fixed common ancestor binding energy

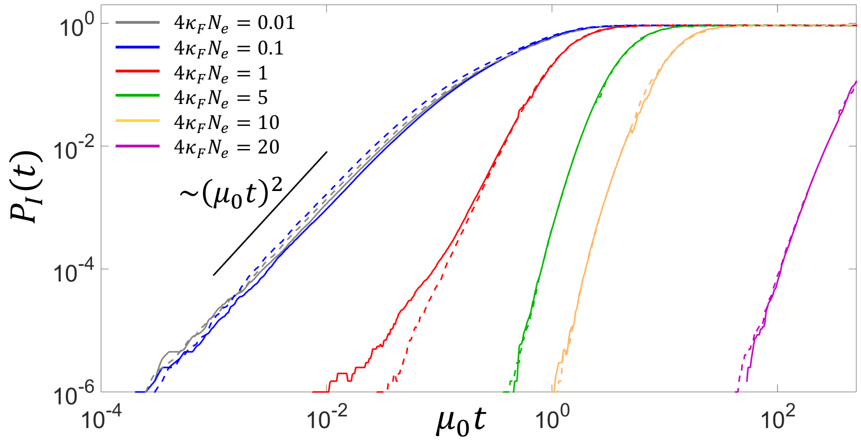

Figure S1 Average probability of a DMI as a function of time after divergence from common ancestor $\mu_0 t$ calculated from simulations for various scaled population sizes, for $\ell = 10$. Solid lines (exactly the same as Fig.1 in main text) correspond to common ancestor sequences drawn from the equilibrium distribution Eqn.7 in main text, while dashed lines correspond to a fixed common ancestor with the mean binding energy at each population size.

We also repeated simulations for the case where replicate runs are performed with the common ancestor sequences always having the mean binding energy from the equilibrium probability distribution (Eqn.7 in main text) at each population size. The results in Fig.S1 show that drawing the common ancestor from the equilibrium distribution is nearly identical to a fixed common ancestor. This suggests that the reason for the power law is related to the fact that the common ancestor is very close to the inviability boundary and so only a small number of substitutions is required for hybrids to become inviable. As discussed in the main text, the distribution of times of substitutions will be Poisson distributed giving a power law for $P_I(t)$ to leading order in $\mu_0 t$, for $\mu_0 t \ll 1$. However, there are some small differences between the two results: 1) for very small population sizes the rate of reproductive isolation is very slightly faster for $4\kappa_F N_e = 0.1$ than $4\kappa_F N_e = 0.01$; 2) for $4\kappa_F N_e = 1$ and for short times, averaging over the equilibrium common ancestor distribution predicts more rapid RI than from a fixed common ancestor. The latter is likely to arise since for $4\kappa_F N_e = 1$ the equilibrium distribution is broad and peaked away from the inviability boundary (Fig.2 main text - red lines) and so the probability of a DMI at short times is dominated by the tail of the distribution closest to the boundary, a phenomenon which cannot happen when when drawing the common ancestor from the mean.

Dynamics of hybrid binding energies

Plotted in Fig.S2 is the average of the hybrid binding energy as a function of scaled population size calculated over 10^4 replicate Gillespie simulations. At zero divergence, the average hybrid binding energies are equal to the average binding energies for that scaled population size, as shown in Fig.2 in the main text. For long divergence times, the hybrid binding becomes weaker, with

[1]The Francis Crick Institute, Mill Hill Laboratory, London, United Kingdom, bhavin.khatri@physics.org

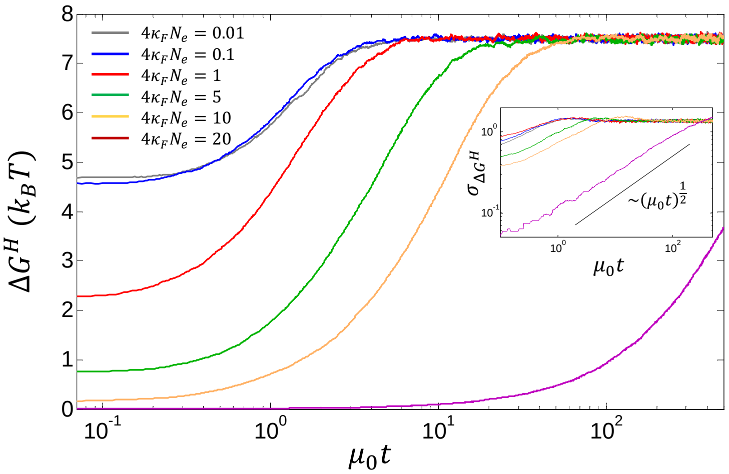

Figure S2 Average hybrid binding energy $\langle \Delta G^H \rangle$ as a function of time after divergence from common ancestor $\mu_0 t$ for $\ell = 10$. The inset shows the root mean square deviation $\sigma_{\Delta G^H} = \sqrt{\langle (\Delta G^H - \langle \Delta G^H \rangle)^2 \rangle}$ of hybrid binding energies as a function of divergence time.

the binding energies increasing to a value $\Delta G^H = 22.5 k_B T$, irrespective of scaled population size, corresponding to the mean of the neutral distribution in Eqn.6 in main text; this is exactly what we would expect after a long period of divergence, as protein and DNA sequences from different lineages should have effectively random interactions. The rate at which this neutral distribution is reached depends strongly on the scaled population size in an approximately monotonic manner, as would be predicted from the average substitution rate seen in Fig.S4. The inset of Fig.S2 shows the root mean square, $\sigma_{\Delta G^H} = \sqrt{\langle (\Delta G^H - \langle \Delta G^H \rangle)^2 \rangle}$ of hybrid binding energies vs $\mu_0 t$ on a log-log scale; we see that in the limit of large scaled population sizes that $\sigma_{\Delta G^H} \sim \sqrt{\mu_0 t}$, suggesting that the underlying dynamics of the hybrids is effectively diffusive.

Dependence of results on sequence length ℓ

Average binding energy on each lineage

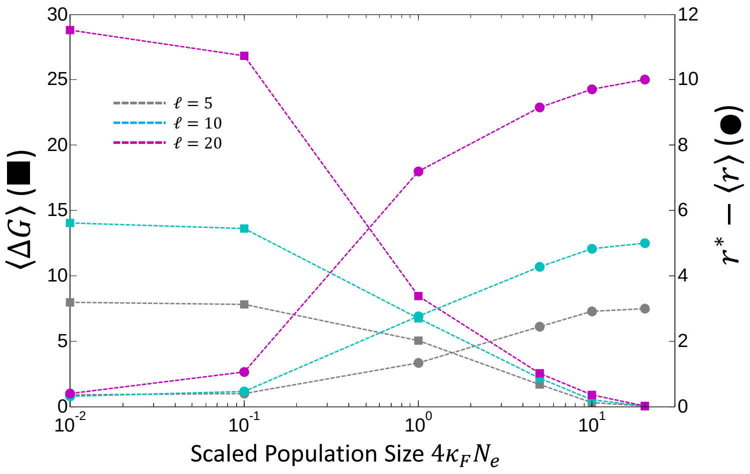

Figure S3 Average binding energy, $\langle \Delta G \rangle = \varepsilon \langle r \rangle$, (left axis, squares) and average Hamming distance of populations from inviability boundary, $r^* - \langle r \rangle$, (right axis, circles) as function of scaled population size $4\kappa_F N_e$ and sequence length ℓ calculated using KMC simulations. We see that as the population size is decreased the mean hamming distance or binding energy ($\sim$ drift load) increases monotonically and towards the inviability boundary.

In the main text Fig.2 showed how the distribution of binding energies changed with scaled population size for a sequence length $\ell = 10$; the figure demonstrated how the drift load increased for decreasing scaled population size. This greater drift load is also illustrated in Fig.S3, which shows the average binding energy and also the Hamming distance of the populations to the inviability

boundary, as a function of the scaled population size $4\kappa_F N$, for sequence lengths $\ell = \{5, 10, 20\}$; for the corresponding values of ℓ, we choose $r^* = \{3, 5, 10\}$, so as to approximately satisfy $r^* = \ell/2$. We see the average binding energy (squares) is larger for smaller population sizes, which corresponds to populations being closer to the inviability boundary as shown by the circles in Fig.S3, and hence also a larger drift load. For large population sizes ($4\kappa_F N_e \gg 1$), where fitness dominates, the drift load is zero, independent of N_e, as $\langle \Delta G \rangle \to 0$. This means that, as shown in Fig.S3, the average Hamming distance to the inviability boundary increases for increasing sequence length – this arises trivially as $r^* \propto \ell$ – however, for small population sizes ($4\kappa_F N_e \ll 1$) the average Hamming distance to the boundary is roughly independent of sequence length. To understand this we consider that for small populations the distribution is neutral and peaked at the inviability boundary $r^*(\ell)$, as shown in Fig.2 of the main text and by the fact the mean binding energy is close to $\Delta G^* = \epsilon r^*$, for $4\kappa_F N \ll 1$ in Fig.S3; at the inviability boundary the number of mutations that increase the Hamming distance is just the number of locations that are matched, multiplied by the number of nucleotides that can give a mismatch, $3(\ell - r^*(\ell)) = 3\ell/2$ and those that decrease it is just the number of mismatched locations, $r^* = \ell/2$. The ratio of these two quantities is independent of ℓ, showing that there is no net drift bias of the populations at the inviability boundary as ℓ changes and so for small populations the average distance to the inviability boundary is roughly independent of ℓ. As we will see the initial distance of the common ancestor from the inviability boundary has a strong impact on the rate of accumulation of DMIs, as functions of population size and sequence length.

Average substitution rate on each lineage

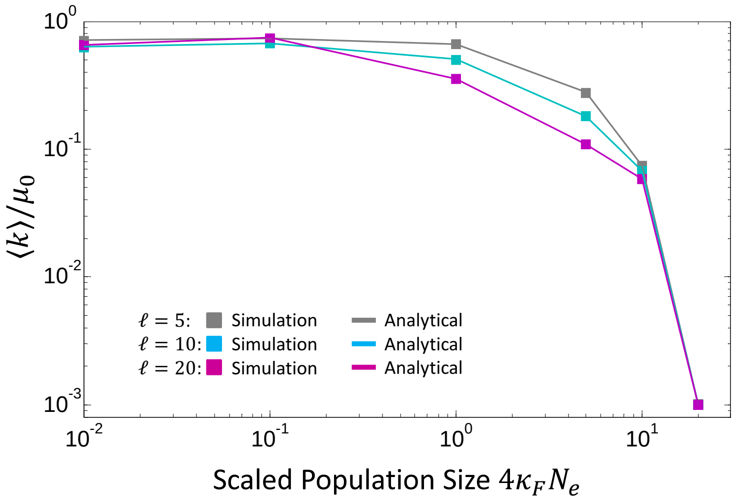

Figure S4 Average total substitution rate for both protein and DNA loci, on a single lineage as function of scaled population size $4\kappa_F N$ and sequence length ℓ. Substitution rate is plotted in units of the nucleotide mutation rate μ_0. The solid circles represent KMC simulations, while the solid lines are the theoretical prediction of the average rate $\langle k \rangle = \frac{2N_e\mu_0}{3\ell} \sum_{r=0}^{r^*} p_\ell(r) \left(r \left(\pi^-(r) + \frac{1}{N_e} \right) + 3(\ell - r)\pi^+(r) \right)$, where $p_\ell(r)$ is the equilibrium distribution of Hamming distances (shown by Eqn. ? in the main text) and π^- and π^+ are the fixation probabilities for the transition $r \to r-1$ and $r \to r+1$, respectively.

In Fig.S4, we find a non-trivial dependence of the substitution rate on sequence length; at large population sizes, as expected, the substitution rate per location is independent of sequence length, but strongly diminished compared to the neutral rate μ_0, as discussed above, due to the discrete changes in fitness being larger than the inverse of the population size. For small populations, we also find that the substitution rate is roughly independent of sequence length; as the distribution of binding energies is peaked at the inviability boundary the substitution rate will be proportional to the number of viable substitutions multiplied by the neutral rate, $\sim \mu_0 r^*(\ell)/\ell = \mu_0/2$, which as observed in Fig.S4 is independent of ℓ. However, for intermediate population sizes, where $4\kappa_F N_e \sim 1$ the average substitution rate decreases with increasing sequence length. In the large and small populations size limits, all substitutions are either non-neutral or neutral, respectively, for $0 \le r \le r^*$. However, for intermediate population sizes the quadratic fitness landscape means there is a critical Hamming distance, $r^*_{eff} \approx (4\kappa_F N_e \epsilon^2)^{-1}$, below which substitutions are effectively neutral ($4N_e|\delta F| \ll 1$) and above are non-neutral ($4N_e|\delta F| \gg 1$). The effective substitution rate will then be roughly $\sim \alpha(\ell)\mu_0 r^*_{eff}/\ell$, where $\alpha(\ell) = \sum_{r=0}^{r^*_{eff}} p_\ell(r)$ is the proportion of time, at equilibrium, spent in the nearly neutral region and r^*_{eff}/ℓ is the fraction of nearly neutral substitutions at r^*_{eff}; we expect that $\alpha(\ell)$ will decrease for increasing ℓ, since we find that $p_\ell(r)$ shifts to larger values of r as ℓ increases (not shown), due to an increased degeneracy pressure, as the sequence length is increased. So together with the fact that the fraction of nearly neutral mutations decreases for increasing ℓ, like r^*_{eff}/ℓ, we see that the average substitution rate is smaller for larger sequence lengths at intermediate population sizes ($4\kappa_F N_e = 1$).
